# Development of Osthole-Loaded Microemulsions as a Prospective Ocular Delivery System for the Treatment of Corneal Neovascularization: In Vitro and In Vivo Assessments

**DOI:** 10.3390/ph16101342

**Published:** 2023-09-22

**Authors:** Yali Zhang, Jingjing Yang, Yinjian Ji, Zhen Liang, Yuwei Wang, Junjie Zhang

**Affiliations:** 1 The First of Clinical Medicine, Henan University of Chinese Medicine, Zhengzhou 450046, China; zyl1638905393@163.com (Y.Z.); jyj17856819199@126.com (Y.J.); 2 Ophthalmology Department, Henan Provincial People’s Hospital, Henan Eye Hospital, Zhengzhou University People’s Hospital, Zhengzhou 450003, China; yangjj9089@163.com (J.Y.); liangzhenxh@126.com (Z.L.)

**Keywords:** osthole, microemulsion, ocular drug delivery system, pharmacokinetic, corneal neovascularization

## Abstract

Osthole (OST), a natural coumarin compound, has shown a significant inhibitory effect on corneal neovascularization (CNV). But, its effect on treating CNV is restricted by its water insolubility. To overcome this limitation, an OST-loaded microemulsion (OST-ME) was created to improve the drug’s therapeutic effect on CNV after topical administration. The OST-ME formulation comprised Capryol-90 (CP-90), Cremophor^®^ EL (EL-35), Transcutol-P (TSP) and water, and sodium hyaluronate (SH) was also included to increase viscosity. The OST-ME had a droplet size of 16.18 ± 0.02 nm and a low polydispersity index (0.09 ± 0.00). In vitro drug release from OST-ME fitted well to the Higuchi release kinetics model. Cytotoxicity assays demonstrated that OST-ME was not notably toxic to human corneal epithelial cells (HCECs), and the formulation had no irritation to rabbit eyes. Ocular pharmacokinetics studies showed that the areas under the concentration–time curves (AUC_0-t_) in the cornea and conjunctiva were 19.74 and 63.96 μg/g*min after the administration of OST-ME, both of which were 28.2- and 102.34-fold higher than those after the administration of OST suspension (OST-Susp). Moreover, OST-ME (0.1%) presented a similar therapeutic effect to commercially available dexamethasone eye drops (0.025%) on CNV in mouse models. In conclusion, the optimized OST-ME exhibited good tolerance and enhanced 28.2- and 102.34-fold bioavailability in the cornea and conjunctiva tissues compared with suspensions in rabbit eyes. The OST-ME is a potential ocular drug delivery for anti-CNV.

## 1. Introduction

The cornea is highly specialized avascular tissue [1], a feature that is required for optical performance. After corneal injury or infection, the new vessels might develop from the corneal limbus, and corneal transparency changes. Thus, loss of vision is the general reason for blindness worldwide. Although transplant operations can improve eyesight, they often fail when neovascularization occurs and result in immune rejection [2]. It has been reported that about 1.4 million people develop corneal neovascularization (CNV) in America, accounting for 4.14% of ocular diseases [3]. The avascularity of the cornea is dependent on the two counterbalancing systems of proangiogenic factors and antiangiogenic factors [4,5]. However, inflammation, chemical burns, infections, nutrition status and other damage factors disrupt the balance of the two systems. Many cytokines, such as vascular endothelial growth factor (VEGF) and matrix metalloproteinase (MMP), have great effects during the formation of CNV. Current clinical pharmacological therapeutics for CNV include anti-inflammatory and immunosuppressive agents and VEGF inhibitors [6]. Among them, topically administered steroids are still a first choice for anti-CNV [7]. However, the frequent application of steroids will lead to some adverse reactions, such as corneal infection, cataracts or glaucoma [8], which makes it urgent to look for a better method to cure CNV.

Osthole (OST) is obtained from many medicinal plants such as *Cnidium monnieri* (L.) Cusson and has been verified to have potential therapeutic applications due to its multiple significant pharmacological activities, including anticancer, antiosteoporotic and antiproliferative effects. Previous studies have also shown that OST not only reduces intraocular pressure [9], but also inhibits CNV [10]. However, its water insolubility (0.63 μg/mL) and low bioavailability significantly restrict its eye applications [11]. Therefore, it is very vital to exploit suitable dosage forms for OST.

In recent years, the OST-loaded drug delivery systems have been extensively studied to enhance their solubility and bioavailability, including injections, inclusion compounds, solid dispersions, gels and other dosage forms, which aimed for systemic administration [12]. However, the limitation of OST was still unaddressed. Recently, an OST-loaded drug delivery system was improved by nano-emulsions and liposomes to promote the therapy of Alzheimer’s disease [13,14]. However, OST-loaded nanocarriers for ocular drug delivery (ODD) have not yet been reported, and some important properties, such as the stability and efficacy of topical ocular administration, remain unresolved in research. Moreover, the eyes are a very complicated tissue, and their unique anatomy restricts the absorption and permeation of most active agents [15]. Thus, ODD remains a challenging task for researchers. The major challenges in ODDs for topical administration are poor corneal permeability and a short residence time on the ocular mucosa [16]. In recent years, lipid-based nanocarrier systems have obtained great attention for ODD systems in ocular diseases and shown numerous benefits, such as enhancing the bioavailability of water insoluble drugs, increasing permeability across ocular tissues, providing sustained drug release and reducing some side effects [16,17]. Microemulsions (MEs) are particularly suitable for ODD, as they overcome many problems, including enhancing residence time and promoting penetration in the corneal. Furthermore, MEs are simple, cheap, easy to produce and sterilize and thermodynamically stable.

The goal of the study was to prepare OST-loaded microemulsion (OST-ME) to treat CNV. The OST-ME was optimized using the experimental statistical design technique (central composite design response surface methodology, CCD-RSM). The OST-ME was characterized by some important parameters, such as droplet size (DS), polydispersity index (PDI), morphology, drug entrapment efficiency (EE) and drug loading (DL). The in vitro drug release from the OST-ME was evaluated. The cytotoxicity of OST-ME was assessed in human corneal epithelial cells (HCECs). Furthermore, we evaluated the therapeutic effect of OST-ME as topical eye drops for anti-CNV in a mouse model, and irritation and the pharmacokinetic profiles in rabbit eyes were also investigated.

## 2. Results

### 2.1. Solubility Study

**Figure 1:**
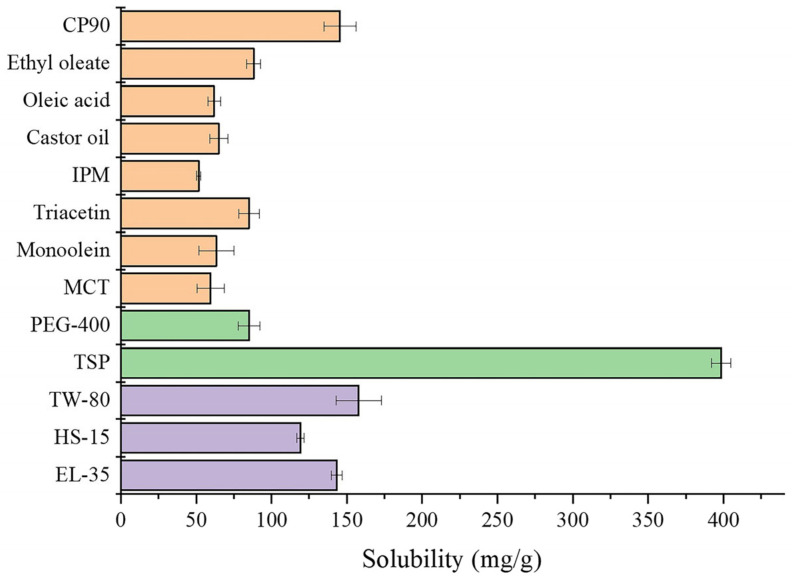
The screen data of OST in oils, surfactants and cosurfactants.

The solubilities of OST in each vehicle are shown in Figure 1. The solubility of OST in Capryol-90 (CP-90, 145.67 ± 10.63 mg/g) was the highest among the oil solvents and that in Transcutol P (TSP, 398.42 ± 6.56 mg/g) as the cosurfactant was much higher than that with the cosurfactant in Polyethylene glycol (PEG400). Thus, CP-90 was chosen as the oil phase, while TSP was chosen as the cosurfactant in the OST-ME formulation. However, the solubilities of OST in Cremophor^®^ EL-35 (EL-35) and Tween-80 (TW-80) were 143.31 ± 3.69 mg/g and 157.90 ± 14.96 mg/g, with no significant difference, so the emulsifying abilities of EL-35 and TW-80 had to be further tested to select a suitable surfactant.

### 2.2. Screening the Emulsification Abilities of TW-80 and EL-35

**Figure 2:**
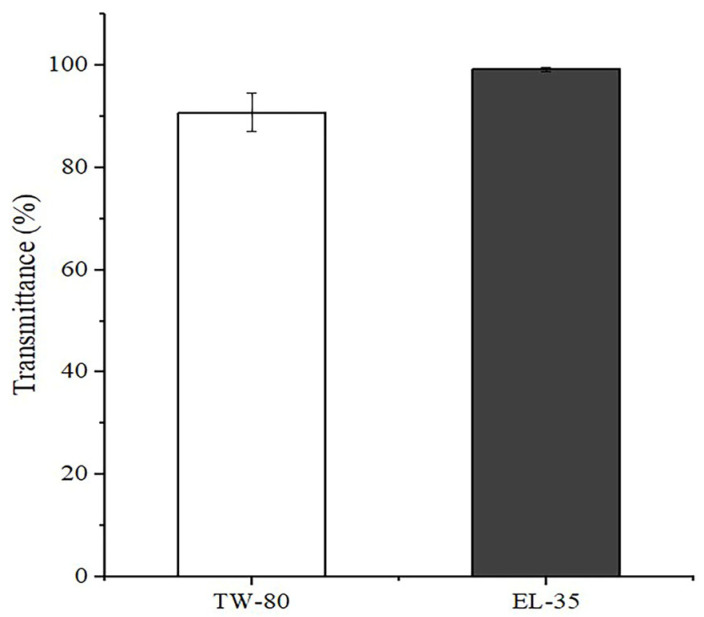
Transmittance of the emulsion consisting of CP-90 and surfactant.

The transmittance values of the emulsions prepared by emulsifying CP-90 with TW-80 and EL-35 were 90.76 ± 3.76% and 99.23 ± 0.39%, respectively, as shown in Figure 2. The transmittance of the emulsion consisting of CP-90 and EL-35 was higher than that of the emulsion consisting of CP-90 and TW-80, which demonstrated that EL-35 had a better ability to emulsify CP-90. Thus, EL-35 was selected as the surfactant.

### 2.3. Construction of Pseudoternary Phase Diagrams

**Figure 3:**
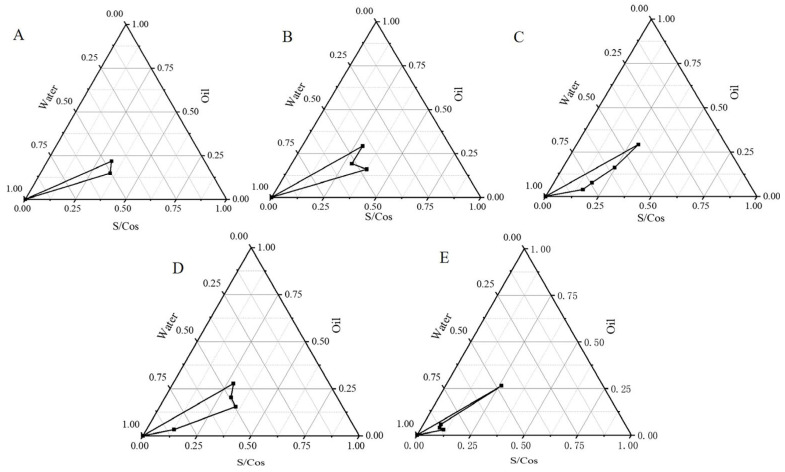
PTPDs of systems with different Km: (**A**) Km = 1:1; (**B**) Km = 2:1; (**C**) Km = 3:1; (**D**) Km = 4:1; (**E**) Km = 5:1.

Pseudo-ternary phase diagrams (PTPDs) were constructed to determine the optimal range of ingredient concentrations. The PTPDs with EL-35 and TSP of different Km values are shown in Figure 3. The MEs’ regions were at a maximum when the Km values were between 2–4. Thus, the range of Km values was 2–4, and the oil concentration was 2–5% in further studies.

### 2.4. Central Composite Design Response Surface Methodology

The independent variables, oil concentration (X_1_) and Km (X_2_), had a significant effect on the response variables, including DS (Y_1_) and PDI (Y_2_). The DS and PDI values from the thirteen runs showed variations from 14.51 nm to 34.67 nm and 0.08 to 0.40, respectively (Table 1). The equations generated for each response are as follows:

Y_1_ = 20.81 − 5.24 X_1_ + 7.03 X_2_ − 0.3475 X_1_X_2_ + 3.23 X_1_^2^ + 1.83 X_2_^2^ − 0.1461 X_1_^2^X_2_ + 7.22 X_1_X_2_^2^ (R^2^ = 0.9961) (1)

Y_2_ = 0.1340 − 0.1096 X_1_ + 0.0707 X_2_ − 0.0300 X_1_X_2_ + 0.0536 X_1_^2^ + 0.0811 X_2_^2^ + 0.0393 X_1_^2^X_2_ + 0.1546 X_1_X_2_^2^ (R^2^ = 0.9977) (2)

**Figure 4:**
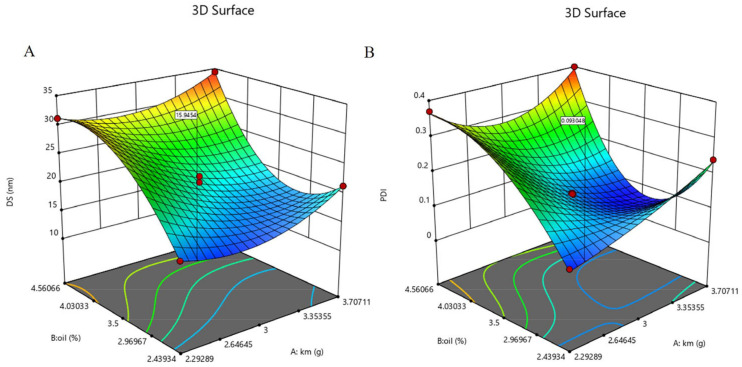
3D response surface plot showing the effect of independent variables on (**A**) DS and (**B**) PDI.

**Figure 5:**
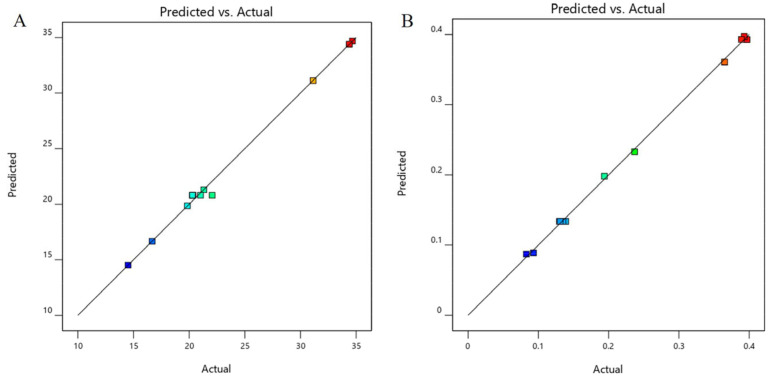
The predicted and actual values of (**A**) DS and (**B**) PDI.

Mathematical Equations (1) and (2) describe the relationship between DS (Y_1_) or PDI (Y_2_) and the independent variables oil concentration and Km, respectively. The coefficients (R^2^) were 0.9961 and 0.9977, respectively, which means that there is a good fit between the independent variables and the DS and PDI. The effect of these independent variables on the DS and PDI of the OST-ME are shown in Figure 4, and the predicted and actual values of DS and PDI are shown in Figure 5.

### 2.5. Preparation and Characterization of the OST-ME

**Figure 6:**
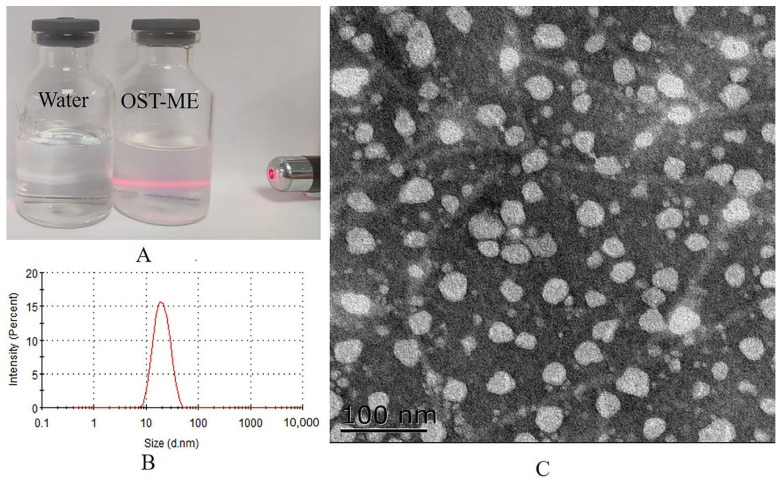
The appearance (**A**), the DS distribution (**B**) and the TEM image (**C**) of the OST-ME (scale bar = 100 nm).

According to the abovementioned optimization experiments, the optimized OST-ME formulation contained 2.44% CP-90, 5.35% EL-35 and 2.21% TSP. Three OST-ME samples were made according to the optimal formulation; they were colorless and had good physical stability. A TEM image, DS (16.18 ± 0.02 nm) and PDI (0.09 ± 0.00) of OST-ME are shown in Figure 6, and other characterization results are listed in Table 2. The EE% and DL% were 99.15 ± 0.66% and 3.70 ± 0.53%, respectively. The osmolarity value was 298.89 ± 1.54 mOsm/kg and the pH value was 6.61 ± 0.99, both of which meet the requirements of eye drops.

### 2.6. Fourier Transform Infrared Spectroscopy Analyses

**Figure 7:**
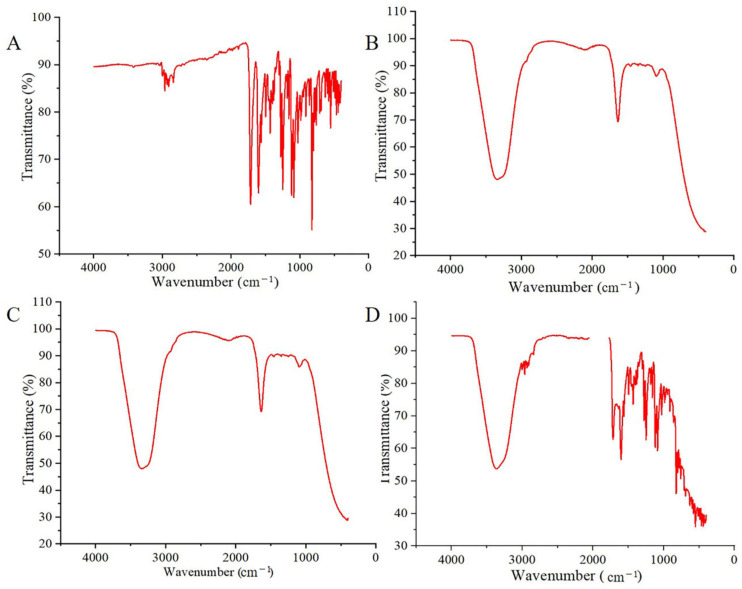
The spectra of pure OST (**A**), blank-ME (**B**), OST-ME (**C**) and PM (**D**).

An Fourier Transform Infrared (FTIR) spectroscopy examination was tested to identify probable interactions between OST and the other excipients during preparation of the ME. The FT-IR spectra of OST, blank ME, OST-ME and the blank ME/OST PM are displayed in Figure 7. The FT-IR spectrum of pure OST has characteristic peaks at 1750 cm^−1^ for C=O stretching, C-H stretching in the range of 2900–3100 cm^−1^, C=C stretching in the 1500–1650 cm^−1^ range and C-O stretching at 1640–1750 cm^−1^. The results are similar to those reported in a previous paper [18]. The FTIR spectrum of the PM showed a combination of the OST and blank-ME individual spectra, but some of the characteristic peaks of OST still existed, indicating that no interaction happened between the pure OST and blank-ME. OST-ME and blank-ME had similar spectra, and no new peaks were observed. The characteristic peak of the pure drug did not appear in the OST-ME spectrum, suggesting that the pure drug was completely dissolved by CP-90 (oil phase). Previous similar results have been reported in the research on luliconazole-loaded nanoemulsions and tacrolimus-loaded microemulsions [19,20].

### 2.7. Short-Term Stability

**Figure 8:**
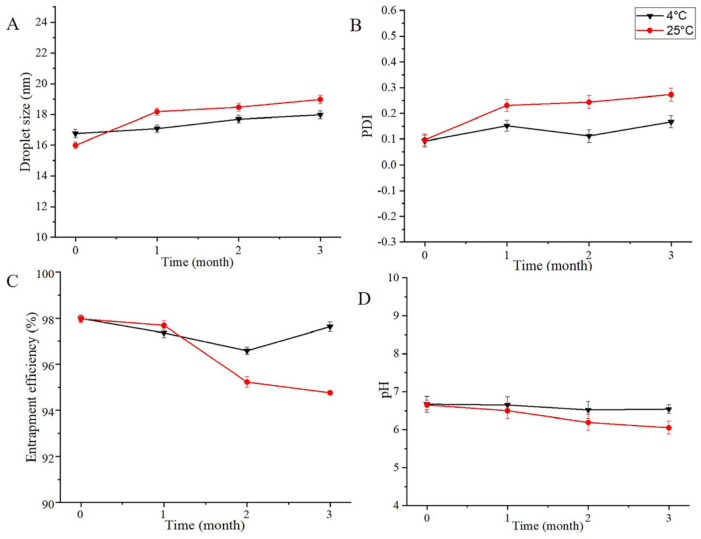
Stability study of OST-ME (**A**) DS, (**B**) PDI, (**C**) EE, (**D**) pH.

The results of the stability investigation are shown in Figure 8. The appearance of the OST-ME was transparent and colorless, which showed that OST was stable in the ME system during the test period. No significant changes in the chemical or physical characteristics (EE, PS, PDI and pH) were observed at 4 °C. However, at 25 °C, both the DS and the PDI showed a slow upward trend; the DS changed from 15.99 ± 0.22 nm to 18.98 ± 0.27 nm, and the PDI increased from 0.10 ± 0.02 to 0.27 ± 0.02. The EE% and pH showed decreasing trends: the EE% decreased from 97.97 ± 0.17% to 94.86 ± 0.09% and the pH value deceased from 6.65 ± 0.13 to 6.05 ± 0.17. Thus, OST-ME could be stable at 4 °C.

### 2.8. In Vitro Drug Release Study

**Figure 9:**
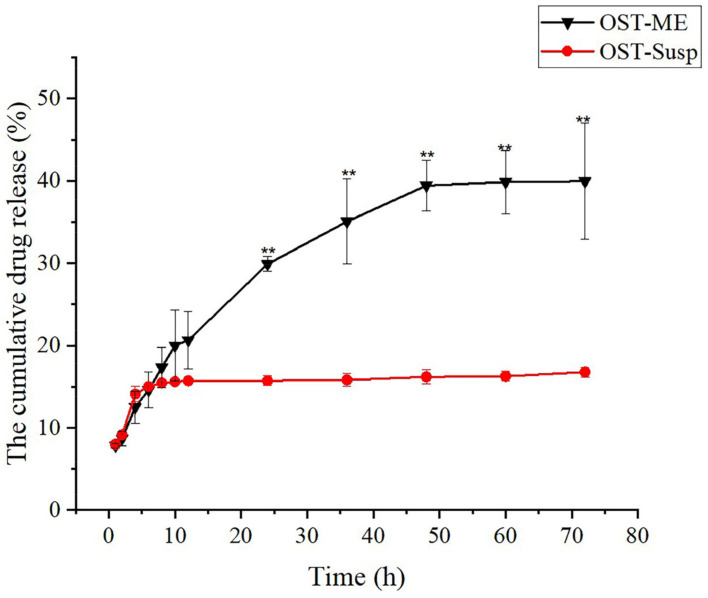
In vitro drug release profiles of OST-ME and OST-Susp. Data represented as mean ± SD, n = 3 (** *p* < 0.01 vs. OST-Susp. Independent samples *t*-test).

The drug release profiles of OST-ME and OST suspension (OST-Susp) in simulated tear fluid (STF, pH 7.4) are shown in Figure 9. The OST-ME had a better sustained release, which was observed at 48 h of dialysis. It is evident that the cumulative release rate of OST-ME was much higher than the OST-Susp (*p* < 0.01). The result shows that the microemulsion-based formulation shows increased OST release compared to the suspensions. The cumulative release percentage of OST-ME is initially rapid, with over 20.01% of the loaded drug being released in the first 10 h. After this phase, there is a steady gradual increase over the next 38 h. The release data were fitted to different dissolution models, including the zero-order, first-order, Higuchi and Korsmeyer–Peppas models, and the Higuchi equation fitted the curve well for the OST-ME (R^2^ = 0.99). While the release percentage of OST-Susp is initially rapid with over 14.12% of the loaded drug being released in the first 4 h, then there is a slight increase over the next 44 h. The Korsmeyer–Peppas equation fitted the curve well for the OST-ME (R^2^ = 0.93). All equation fitting and correlation coefficient results are listed in Table 3.

### 2.9. In vitro Cell Viability Study

**Figure 10:**
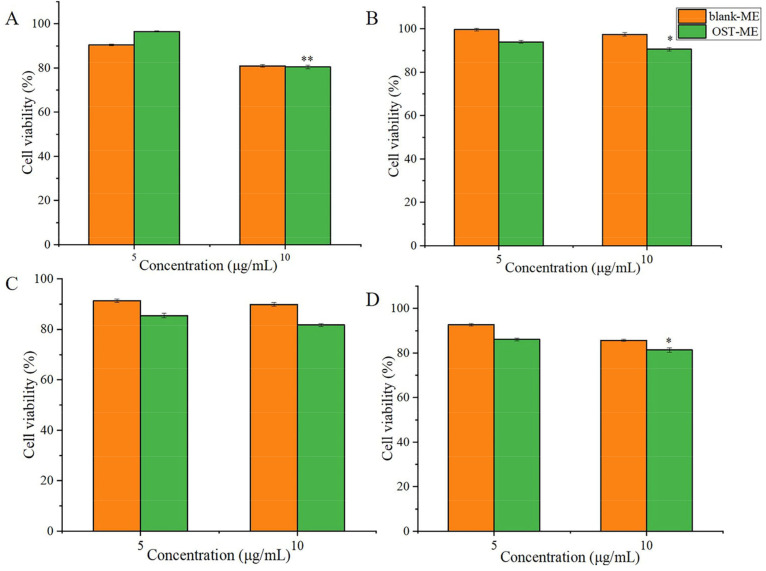
Histogram of the percentage cell viability of OST-ME. Results of CCK-8 assay at incubation times of (**A**) 0.25 h, (**B**) 1 h, (**C**) 2 h and (**D**) 4 h with blank-ME and OST-ME (5 μg/mL, 10 μg/mL). * *p* < 0.05, ** *p* < 0.01 vs. 5 μg/mL OST-ME. Independent samples *t*-test.

The cell toxicity of OST-ME was evaluated by the CCK-8 assay. The cytotoxicity profiles of the OST-ME and blank-ME after incubation at different time points are shown in Figure 10A–D, respectively. The figure demonstrates that OST-ME is safe for HCECs after different durations of incubation, as indicated by the high cell viability percentage (>80%).

### 2.10. The Ocular Irritation Test

**Figure 11:**
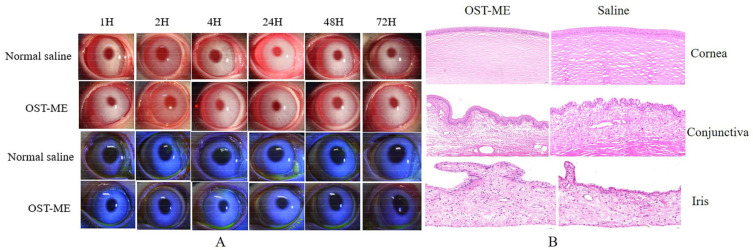
Ocular irritation result (**A**) and histopathological analysis of cornea, iris and conjunctiva (**B**).

The study was tested with rabbits according to the modified Draize test. No obvious ocular irritancy symptoms were detected in the test or control eyes. The scores in terms of the cornea, conjunctiva, redness, discharge and iris alterations were 0 in all rabbits. Moreover, the image of the irritation result was photographed by the sodium fluorescein (Figure 11A) [21]. Hematoxylin and eosin (H&E) stained tissue sections were used to assess cell structure and tissue integrity. Representative micrographs of three types of tissues (cornea, conjunctiva and iris) treated with OST-ME and saline are shown in Figure 11B. The figure of the cornea, conjunctiva and iris showed smooth and clear tissue structures in the experiment group and control group. These results illustrate that OST-ME meets the needs of safety and is nonirritating, which suggests suitability for ocular drug delivery.

### 2.11. Ocular Pharmacokinetics Study

**Figure 12:**
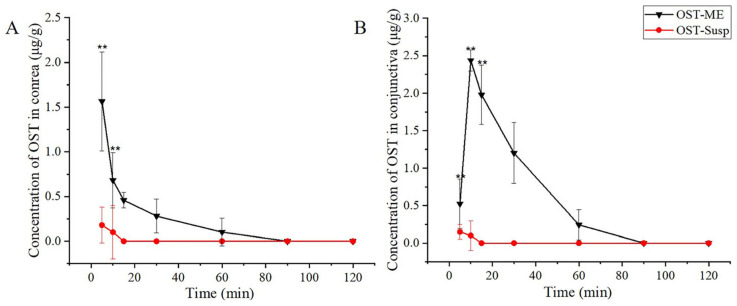
Concentration-time curves of OST in rabbit cornea (**A**) and conjunctiva (**B**) (** *p* < 0.01 vs. OST-Susp).

The distributions of OST in the corneal and conjunctival tissue are shown in Figure 12, and the ocular pharmacokinetics parameters are shown in Table 4. The OST concentrations in the corneal and conjunctival tissue were much greater in the OST-ME group than in the OST-Susp group after a single administration. Moreover, the levels of OST in the OST-Susp group declined to below the limit of quantitation (LOQ, 0.025 μg/mL) 10 min and 15 min after administration. The AUC_0–t_ value of the corneal and conjunctival tissue in the OST-ME group were 19.74 and 63.96 μg/g·min, respectively. Therefore, the ME displayed a notable advantage over OST suspension in increasing the permeability of OST across the cornea and conjunctiva.

### 2.12. In Vivo Anti-CNV Efficacy in Mice

#### The Image and Area of CNV

**Figure 13:**
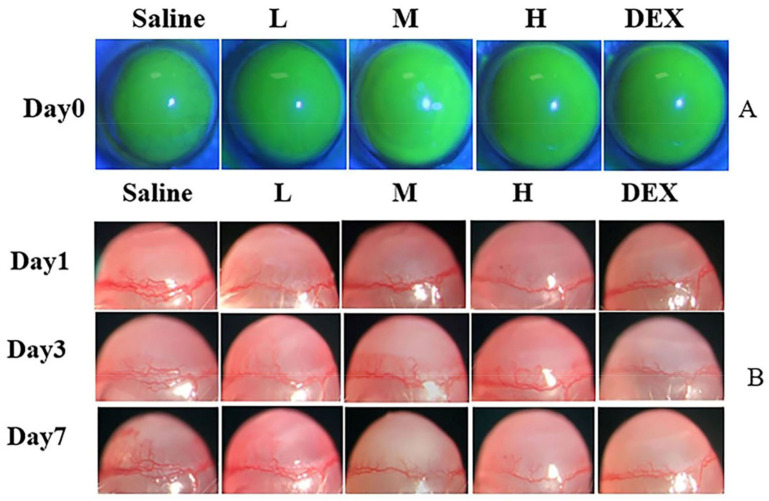
The fluorescein sodium images after modeling on day 0 (**A**) and slit lamp images of CNV in different groups on days 1, 3 and 7 (**B**).

**Figure 14:**
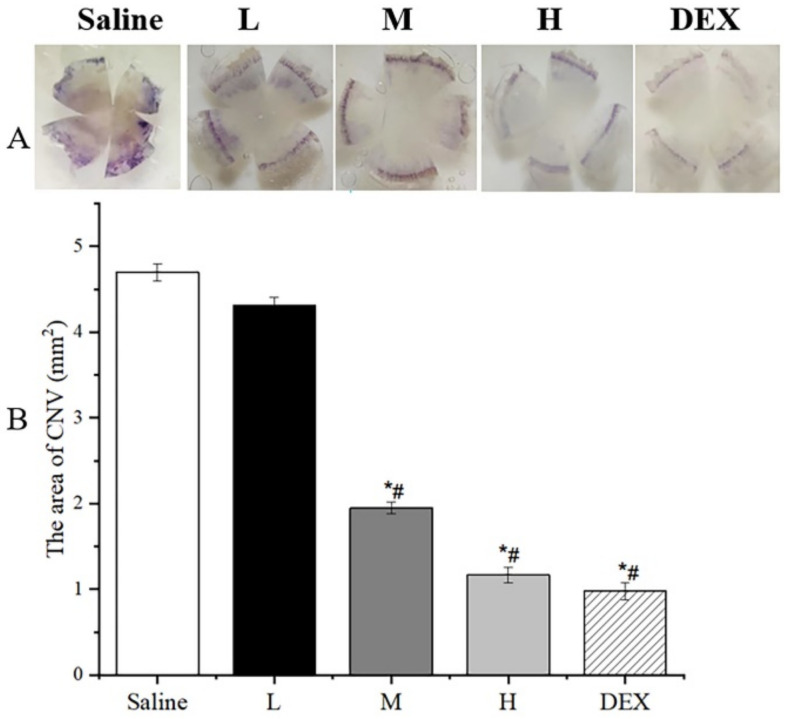
(**A**) Hematoxylin staining images of different groups on day 7. (**B**) The area of CNV 7 days after alkali burn (* *p* < 0.05 vs. saline group; # *p* < 0.05 vs. L group).

The anti-CNV effect of OST-ME was evaluated. As shown in Figure 13A, the burn areas of the epitheliums were similar among the groups, and there was no significant difference. To observe whether OST-ME can restrain CNV, the images (Figure 13B) were taken by microscope camera system. After treatment on day 1, CNV had already grown from the corneal limbus in all groups. The CNV showed an increasing trend on days 3 and 7 (Figure 13B) in the saline treatment group. On day 3 after treatment, although CNV had continued the growth trend, CNV in the 0.1% OST-ME (M), 0.2% OST-ME (H) and Dexamethasone (DEX) groups had shorter and thinner new vessels than that in the 0.05% OST-ME (L) and saline groups. On day 7 after treatment, the CNV of the M (0.1% OST-ME) and H (0.2% OST-ME) groups was reduced (Figure 14A). The areas of the saline group were significantly higher than other groups (*p* < 0.05) (Figure 14B), but there was no significant difference between group M and H (*p* > 0.05). These results demonstrated the OST-ME can restrain CNV.

### 2.13. Histopathological Examination

**Figure 15:**
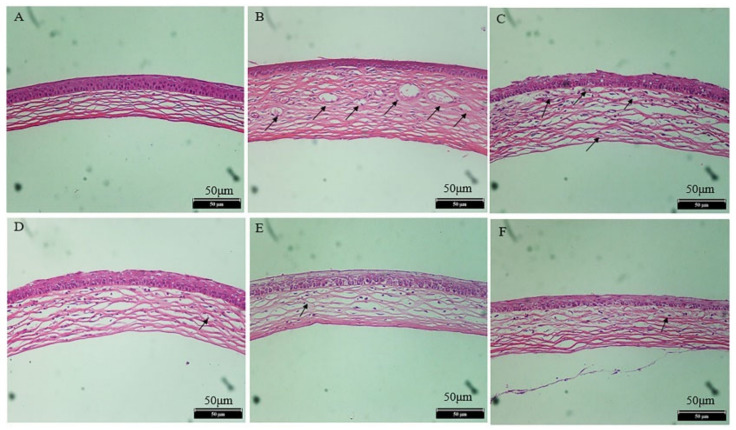
H&E staining of cornea (x 200): (**A**) the normal group; (**B**) the saline group; (**C**) the 0.05% OST-ME group; (**D**) the 0.1% OST-ME group; (**E**) the 0.2% OST-ME group; (**F**) the DEX group. The black arrow indicates cornea neovascularization.

The H&E section was used to evaluate the structural difference among every group. As illustrated in Figure 15A, the normal cornea showed orderly arranged epithelial cells and no structural or pathological changes in the vasculature. In Figure 15B, the epithelial cells in the saline group were arranged in an orderly manner, but irregular collagen fibers and an enlarged fiber space in the stroma were observed, and new vasculature existed in the corneal structure. Compared with the normal group (Figure 15B), the pathological conditions between the L and M groups (Figure 15C,D) were notably improved to some extent. The epithelial cells and collagen fibers in the stroma between the H and DEX groups were more remarkably improved, and angiogenesis was notably reduced (Figure 15E,F). The result illustrated that the M and H doses of OST-ME can restrain CNV.

### 2.14. Enzyme Linked Immunosorbent Assay

**Figure 16:**
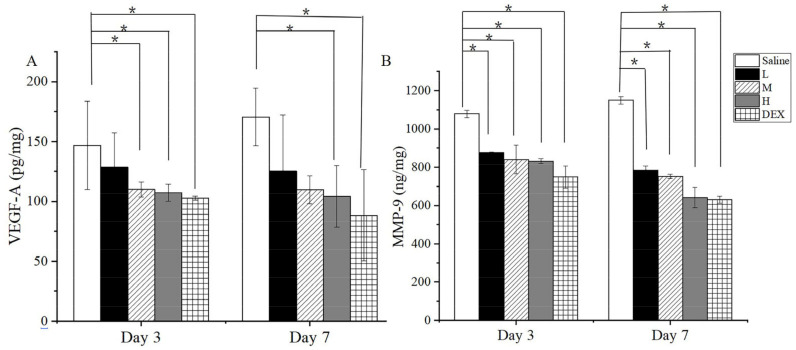
The concentration of VEGF-A (**A**) and MMP-9 (**B**) in corneal tissues at day 3 and day 7 (* *p* < 0.05 vs. saline group).

To investigate the effect of OST-ME on protein expression in CNV, the concentrations of VEGF-A and MMP-9 among total protein were assessed by ELISA. On days 3 and 7, the levels of VEGF-A and MMP-9 in group M, H and DEX were notable lower than those in the saline group (*p* < 0.05) after administration of the corresponding drug. The result illustrated that OST-ME could restrain the level of VEGF-A and MMP-9 in the CNV model (Figure 16).

## 3. Discussion

Previous studies have shown that some pro-angiogenic cytokines, such as VEGF and MMP, would increase in animal models of CNV [22,23,24]. It has been reported that OST, a new antiangiogenic drug, could inhibit the overexpression of VEGF and MMP [25,26], and topical administration of OST could inhibit CNV established by alkali burns in mice [10]. However, the poor water solubility of OST (0.63 μg/mL) limits its clinical application in ophthalmology [11]. MEs, as one of the most prospective forms of nano-carriers for drug delivery systems, have shown some advantages over traditional eye drops for ocular applications [27]. It has been reported that using MEs as vehicles results in good store stability, good corneal permeation, sustained drug release and ultimately increased drug bioavailability in the eye [28,29]. In the present investigation, an oil-in-water (O/W) ME loaded with OST was successfully produced and showed good in vitro and in vivo characteristics.

All inactive ingredients selected for use in the OST-ME formulations, such as CP-90, EL-35, TSP and SH, were included in the FDA Inactive Ingredients Database, and no organic solvents were used in the preparation process, which could avoid potential toxicity due to organic residue. The three excipients are safe and harmless and have already been approved in Europe for topical applications [30], and were thus selected for use in ophthalmic preparation in this study. CP-90 and TSP were selected as the oil phase and cosurfactant because of the high solubility of OST in these solvents. EL-35 was selected as the surfactant, as it had a stronger ability to emulsify the oil phase than TW-80. Moreover, EL-35 and TSP are less affected by pH changes and have been widely applied to improve the bioavailability of insoluble drugs [31].

The PTPDs provided useful information on various ME compositions. The larger the regional area of the ME in the PTPDs, the stronger the ability to form ME [32]. The formulation was optimized based on the PTPDs and CCD-RSM. The response surface plot relationship of the independent variable to DS and PDI is shown in Figure 4. A nonlinear mathematical model was used to fit the result by CCD-RSM. The optimized OST-ME with a small DS was obtained. Figure 4A shows that the DS decreased as the amount of oil and Km decreased. Figure 4B shows that the PDI also decreased as the amount of oil and Km decreased.

In vitro drug release has a significant effect on predicting and understanding the nature of a formulation. The drug release curve of the OST-ME and OST-Susp were better fitted to the Higuchi and first-order equations, and the R^2^ values are 0.99 and 0.94, respectively. The cumulative drug release of the OST-ME was higher than the OST-Susp over 96 h. These data illustrated that the ME has a better ability to release drugs. These results also gave a value of n of 0.42 (n < 0.43), which illustrated that OST release from OST-ME was dependent on Fickian diffusion [33]. Although in vitro drug release experiments provide important information for in vivo experiments, they cannot replace data obtained from in vivo studies.

When ophthalmic drug delivery systems for topical application are developed, ocular safety is always a critical issue. The Draize eye test is a commonly used way to assess the irritation of eye drops and it was used to assess the irritation caused by OST-ME in this study [34]. Here, the eye irritation scores were both 0 in the experiment group and control group, indicating no eye irritation and suggesting good biocompatibility and tolerance. In this study, HCECs were also used to evaluate cell toxicity, and the results illustrated that the OST-ME has good cytocompatibility at proper concentrations.

The pharmacokinetic study was conducted in rabbit eyes. This study had strengths and weaknesses and could directly evaluate the drug concentration in the corneal and conjunctiva tissue. However, since cornea and conjunctiva tissue are not consecutively sampled, it is impossible to harvest tissues from the same animal at every time point. So, the drug level will be impacted by individual absorption and metabolism to some extent [35]. Compared to OST-Susp, OST-ME rapidly penetrated the cornea and conjunctiva due to its ultrasmall particle size, which allowed the material to penetrate into the tissue via the transcellular pathway. It has been reported that ex vivo silicate nanoparticles with a particle size of 40 nm can penetrate bovine corneas [36]. Moreover, some surfactants could also decrease interfacial surface tension and increase the drug permeability across epithelial cells [37]. The conjunctival tissue is more permeable to drugs than the corneal tissue [38], and the drug level in the corneal and conjunctival is affected by individual metabolism [35]. Nevertheless, conjunctival drug absorption was deemed useless due to its distribution by the capillaries and lymphatics into systemic circulation [39]. Correspondingly, in our investigation, OST was undetected in the aqueous humor. The charge and size of a drug and many other factors could influence its permeation across eye barriers [38]. Therefore, the corneal penetration mechanism still needs to be further studied.

Various causes can result in CNV, but chemical injury is clinically one of the most common. In this experiment, an alkali burn injury model was used to induce corneal angiogenesis and evaluate the treatment effect of OST-ME. The results demonstrated that the OST-ME (H) treatment could significantly inhibit CNV when the eye drop was instilled four times a day. The antiangiogenic effects of OST-ME were verified by evaluating the area of CNV (Figure 14B) and H&E-stained sections at each time point (Figure 15). Additionally, 0.1% OST-ME can restrain the level of VEGF-A and MMP-9 in the cornea total protein concentration. It has also been reported that MMP-9 and MMP-2 could play a very important role in CNV [40]. Our results indicated that OST-ME could significantly inhibit the expression of MMP-9. Other researchers have also shown that CNV can be effectively inhibited by topically administering OST suspensions in a carboxymethylcellulose sodium (CMC-Na) solution [10]. The test group, which was treated with a high concentration of the OST-ME, produced results that were not different from those in the DEX group in our investigation, suggesting that OST is a potential alternative to DEX for an anti-CNV drug.

## 4. Materials and Methods

### 4.1. Materials

Osthole (OST), isopropyl myristate (IPM), and monoolein were provided by Macklin Biochemical Co., Ltd. (Shanghai, China). Medium-chain triglycerides (MCTs) were purchased from Yunhong Chemical Preparation Excipients Technology Co., Ltd. (Shanghai, China). Ethyl oleate and triacetin were obtained from J&K Scientific Technology Co., Ltd. (Beijing, China). Oleic acid was provided by Tokyo Chemical Industries Co., Ltd. (Tokyo, Japan). Propylene glycol dicaprylate (PGD) and castor oil were provided by Hunan Er-Kang Pharmaceutical Co., Ltd. (Changsha, China). PEG400 was obtained from Solarbio Life Science (Beijing, China). EL-35 was obtained from Shanghai Puzhen Biotechnology Co., Ltd. (Shanghai, China). Kolliphor HS-15 (HS-15) was obtained from BASF SE (Ludwigshafen, Germany). Tween-80 (TW-80) was obtained from Sichuan Jinshan Pharmaceutical Co., Ltd. (Guangyuan, China). TSP and CP-90 were acquired from Gattefosse (Saint-Priest, France). Glycerin was provided by Huikang Pharmaceutical Co., Ltd. (Lishui, Zhejiang, China). Sodium hyaluronate (SH) was obtained from Furuida Biochemical Co., Ltd. (Qingdao, Shandong, China). A Cell Counting Kit-8 (CCK-8) was acquired from APExBIO (Houston, TX, USA). Mice MMP-9 and VEGF-A enzyme linked immunosorbent assay (ELISA) kits were acquired from Elabscience Biotechnology (Wuhan, China). Dexamethasone (DEX) sodium phosphate solution were provided by Huaqing Pharmaceutical Co., Ltd. (Xinxiang, China).

### 4.2. Animals

New Zealand white rabbits (2.0–2.5 kg) and male BALB/c mice (6–8 weeks old, 20 ± 2 g) were obtained from Huaxing Experimental Animal Breeding Co. (Zhengzhou, China). All rabbits and mice were provided a standard daily diet and water and placed in lab that was kept at 23 °C ± 2 °C with a 12 h light/12 h dark cycle. Every animal was experimented on according to the principle of the Association for Research in Vision and Ophthalmology (ARVO) declaration.

### 4.3. OST Assay by High Performance Liquid Chromatography

The OST concentration was measured by the high performance liquid chromatography (HPLC) method according to a previous report [41,42]. Briefly, the HPLC system (Milford, MA, USA) was equipped with a Waters Symmetry^®^ C18 column (4.6 × 250 mm, 5μm), and the column temperature was set at 40 °C. The mobile phase was made up of methanol–water (85:15, *v*/*v*) under an isocratic conditions flow rate of 1.0 mL/min and the injection volume was 10 μL. The ultraviolet (UV) detector wavelength was 322 nm. The OST concentration was determined from the standard curve.

### 4.4. Solubility Study

To obtain suitable ingredients for the preparation of OST-MEs, the solubilities of OST in different oils (CP-90, ethyl oleate, oleic acid, castor oil, IPM, triacetin, monoolein and MCTs), surfactants (EL-35, TW-80 and HS-15) and cosurfactants (PEG400 and TSP) were investigated according to a previous report [43,44]. All undissolved drugs were removed from the samples, and the samples were diluted with methanol and assayed using a UV-visible spectrophotometer (UV1800SPC, Shanghai, China) at 322 nm [45]. Each experiment was prepared in triplicate.

### 4.5. Emulsification Ability

The ability of the selected surfactant to emulsify the oil phase was assessed according to a previous report [46]. Emulsions transmittance was tested at 650 nm using a UV, taking purified water as a blank control group. Each sample was determined in triplicate.

### 4.6. Pseudo-Ternary Phase Diagrams

The aforementioned three ingredients (oil phase, surfactant and cosurfactant) were chosen to constitute the PTPDs by the water titration phase inversion emulsification (PIE) method according to a previous report [47]. The area had a low viscosity and the transparency appearance was deemed as the ME. The weight percents of the oil, S/Cos and water were recorded and calculated. Then, PTPD was prepared by Origin software (Version 8.0, Northampton, MA, USA).

### 4.7. Optimization of the OST-ME Formulation

The OST-ME formulation was optimized by the experimental statistical design technique with the Design Expert^®^ software (V 12.0.1.0, Minneapolis, MN, USA). CCD-RSM was applied to investigate the effects of independent variables (X_1_: oil concentration, %; and X_2_: Km range) and their interaction over the dependent variables (Y_1_: DS; and Y_2_: PDI) [48,49]. The two-variable relations are shown in Table 5.

### 4.8. Preparation of the OST-ME

The optimal OST-ME formulation could be obtained from the CCD-RSM optimization results and was prepared using the PIE method. Briefly, three selected ingredients were weighed and homogenously mixed at 37 °C. Then, OST was added in the homogenous mixture and dissolved under magnetic stirring. Then, distilled water was added in the mixture until the transparent light blue solution was obtained. Lastly, 10 mL of 0.2% (*w*/*v*) SH solution was added to the OST-ME solution, and the total volume of the mixed solution was 20 mL. The OST-ME was filtered using a 0.22 µm filter. Each sample was prepared in triplicate.

### 4.9. Characterization of the OST-ME

#### 4.9.1. Determination of Droplet Size, Zeta Potential and Polydispersity Index

The average DS, ZP and PDI values of the OST-ME were tested by dynamic light scattering (Zetasizer, NanoZS90, Worcestershire, UK). All samples were diluted (1:20, *v*/*v*) with purified water, and each experiment was performed in triplicate.

#### 4.9.2. Assessment of Entrapment Efficiency and Drug Loading

To calculate the EE% and DL% of OST-ME, 3 mL of OST-ME was added to centrifugal filter tubes (Amicon Ultra4, Ireland Regenerated Celluloses, MWCO: 10 kD) to separate the free OST from the OST-ME by centrifuge (Centrifuge 5810 R, Eppendorf, Germany). All samples were assayed by the aforementioned HPLC method. The value was calculated by the following equations:

(3) $$EE\%=\frac{W_{t}-W_{f}}{W_{t}}\times 100$$

(4) $$DL\%=\frac{Weight\;of\;the\;drug\;in\;the\;ME}{Weight\;of\;the\;drug\;and\;nanocarrier}\times 100$$

where W_t_ is the weight of the OST in the ME and W_f_ is the weight of the free drug in the ME.

### 4.10. Morphological Observations of the OST-ME

The morphology of the optimized OST-ME was observed by transmission electron microscopy (TEM) (Philips Company, Holland Tecnai 12, Eindhoven, The Netherlands). Briefly, OST-ME was diluted 50-fold with purified water before the TEM study. Then, the diluted OST-ME was applied to a carbon-coated copper grid. After drying, OST-ME samples were observed and photographed by TEM at 25 °C.

### 4.11. pH and Osmotic Pressure

The pH and osmolality of the OST-ME were tested by pH meter and freezing point osmometer (STY-A, Tianjin, China). Each experiment was conducted in triplicate.

### 4.12. Fourier Transform Infrared Spectroscopy Analyses

In this study, the OST, Blank-ME, OST-ME and the physical mixture (PM) of 20 mL of Blank-ME/OST (each experimental sample contained 20 mg of OST) were characterized by an AVATAR 370 FTIR instrument (Alpha II, Bruker, Germany). All samples were measured at 25 °C in the range of 4000–400 cm^−1^ [50].

### 4.13. Short-Term Stability

The stability of the optimized OST-ME was assessed for three months. Briefly, the OST-ME was prepared by filtration sterilization, and five milliliters of the sample was loaded in sterilized polypropylene eye drop bottles, sealed with a cover under a cleaning bench and then placed at 4 °C and 25 °C. Both the physical and chemical stabilities of OST-ME formulations were evaluated. All tests were performed on day 0 and at 1, 2 and 3 months, and samples were analyzed in triplicate.

### 4.14. In vitro Drug Release

The drug release of OST was conducted via dialysis in simulated tear fluid (STF, pH 7.4), containing 0.1% TW-80 as the solubilizer, which served as the release medium. The release of OST was analyzed by HPLC, and the cumulative release percentage (Q) of OST was calculated at relevant time points (according to Equation (3)). The Q was fitted to the zero-order kinetics, first-order kinetics, Higuchi and Korsmeyer–Peppas models, and the release curves were drawn using Origin software (Version 8.0, Northampton, MA, USA). The OST-Susp was chosen as the control.

(5) $$Q\%=\frac{C_{n}V+V_{i}\sum_{i=0}^{i=n}C_{i}}{W_{0}}\times 100$$

Here, W_0_ is the total weight of the OST in the OST-ME, C_n_ is the OST concentration in STF at t_n_, V is the total volume of STF, V_i_ and C_i_ are the sample volume and concentration at t_i,_ respectively, and t_n_ is the nth sampling time [51].

### 4.15. Cytotoxicity

The toxicity of OST-ME to HCECs was evaluated with CCK-8 assays. HCECs at a density of 1 × 10^4^ cells per well were exposed to different concentrations of OST-ME (5 μg/mL and 10 μg/mL) and corresponding concentrations of blank-ME, which were cultured for 0.25, 1, 2 and 4 h. Then, 10% CCK-8 (100 μL) was added to every well and cultured for 4 h, and the optical density (OD) of all samples was tested at 450 nm with a microplate reader (PerkinElmer 2104 Multilabel Reader, Shanghai, China). All results were calculated by Equation (4) [52].

(6) $$Cell\;viability\%=\frac{{OD}_{Sample}-{OD}_{blank}}{{OD}_{control}-{OD}_{blank}}\times 100$$

### 4.16. Ocular Irritation Test

The irritation of the OST-ME was measured by six white rabbits according to the Draize eye test [53,54]. None of the eyes of any rabbits had eye disease. A quantity of 100 µL of the OST-ME was instilled into the right eye, while the contralateral eye used 0.9% saline solution as a control. Both eyes of all rabbits were examined, scored and recorded for signs of ocular irritation after administration of 1, 2, 4, 24, 48 and 72 h. The results were recorded according to the Draize technique [34]. Furthermore, the rabbits were euthanized via an injection of 4% pentobarbital sodium solution through the ear vein 72 h after exposure. The eyeballs were dissected and fixed with FAS, and after staining with H&E, histopathological observation of the corneal, conjunctival and iris was performed using an optical microscope (Nikon 80i, Nikon Corporation, Tokyo, Japan) [55].

### 4.17. Ocular Pharmacokinetics

#### 4.17.1. Grouping and Dosing

Forty-two healthy rabbits were randomly divided into two groups (test group and control group, 21 rabbits in each group), which were randomly divided into seven subgroups (n = 3), respectively. Both eyes of each animal were instilled with 50 μL of 0.1% OST-ME in the test group and the OST-Susp in the control group. The animals were euthanized by an injection of 4% phenobarbital sodium solution at scheduled time points. The corneal and conjunctival tissues were collected. Then, the tissues were weighed and stored at −80 °C for HPLC analysis. The drug in the tissue samples was extracted according to a previous report [56].

#### 4.17.2. Analysis of Ocular Tissues

A validated method reported in a previous study was revised to gain OST from corneal and conjunctival tissue [57]. Briefly, the harvested conjunctiva and cornea samples were cut into pieces and soaked in methanol (0.4 mL) and then stored at 4 °C for 24 h. All samples were centrifuged (12,000 rpm, 10 min) and the supernatant was drawn and determined by the abovementioned HPLC method. Corneal and conjunctival tissue working solutions were acquired by diluting the standard solutions with corresponding blank rabbit tissues in order to prepare the rabbit tissue calibration curves. The pharmacokinetic parameters were obtained using DAS2.1.1 software (Anhui Provincial Center for Drug Clinical Evaluation, Wuhu, China).

### 4.18. Anti-CNV Study

#### Establishment of CNV Model

The CNV mouse model was established using the alkali burn method described in a previous report [58]. After one day, CNV was imaged by slit lamp microscope. Then, eighty mice were randomly divided into five groups (n = 16). Every mouse in each group was instilled with 5 μL of saline (saline group) or the same volume OST-ME (L, 0.05%, *w*/*v*), OST-ME (M, 0.1%, *w*/*v*), OST-ME (H, 0.2%, *w*/*v*) or dexamethasone (DEX, 0.025%, *w*/*v*). Each group was treated four times a day for a total of 7 days.

### 4.19. Assessment and Quantification of CNV Area

After treatment days 1, 3 and 7, CNV images of all animals were taken by microscope [58]. To quantitatively analyze the CNV area, a corneal flat mount method was used. On day 7, aortic perfusion was performed on three mice in each group. Then, the right eye of each mouse was enucleated and fixed in FAS at 4 °C, after which the corneal tissue was dissected and flattened for taking imaging. The area was calculated with the following Equation (5) [59,60].

(7) $$A=C/12\times 3.1416\times \left[r^{2}-{\left(r-L\right)}^{2}\right]$$

Here, A is the area, C is the number of clock hours of CNV that were involved, L is the length of the new blood vessel and r is the corneal radius [61].

### 4.20. Histopathological Examination

After day 7, three mice were sacrificed and the whole eyeball was enucleated from each. The corneas were separated and fixed in FAS trimming for hematoxylin-eosin (H&E) staining and histopathological observations.

### 4.21. Enzyme Linked Immunosorbent Assay

The expression levels of VEGF-A and MMP-9 in the corneas were measured. After days 3 and 7, five mice were sacrificed in each group, and the corneal samples were dissected and saved at −80 °C until analysis. The corneal samples were rewarmed at 4 °C for half an hour before analysis. The samples were treated according to the previous method [58]. The concentration of VEGF-A and MMP-9 in the corneas was measured according to the manufacturers’ instructions. All samples of the OD were tested by a microplate reader at 450 nm.

### 4.22. Statistical Analysis

All results were analyzed by SPSS software (SPSS version 21, Chicago, IL, USA). Fisher’s least significant difference (LSD) test was recorded to compare the differences between every group; *p* < 0.05 was considered as statistical significance. All data were recorded as the mean ± standard deviation (SD).

## 5. Conclusions

In this study, an optimized OST-ME formulation was successfully generated and characterized. The formulation showed good tolerance. Moreover, the formulation had good storage stability at 4 °C. In vitro drug release studies illustrated that OST-ME had a better sustained drug release rate than OST-Susp. In vivo pharmacokinetic studies illustrated that OST-ME had better bioavailability than OST-Susp and that the retention time of OST-ME on the cornea was prolonged. In addition, OST-ME effectively inhibited CNV and decreased VEGF-A and MMP-9 protein expression. In summary, OST-ME could be a potential drug for anti-CNV.

## Figures and Tables

**Table 1:** The factors and levels of CCD-RSM (X_1_: oil concentration; X_2_: Km; Y_1_: DS; Y_2_: PDI).

Independent Variables			Value of Response			
			Actual Value		Predicted Value	
Formulation	X_1_(%)	X_2_	Y_1_ (nm)	Y_2_	Y_1_ (nm)	Y_2_
1	2	3	14.51	0.194	14.31	0.199
2	4.56066	3.70711	34.39	0.397	34.92	0.397
3	4.56066	2.29289	31.14	0.369	31.93	0.362
4	3.5	3	22.07	0.142	21.97	0.148
5	3.5	3	20.30	0.134	20.07	0.131
6	3.5	3	20.36	0.139	20.01	0.135
7	2.43934	2.29289	16.68	0.093	17.22	0.095
8	3.5	2	34.67	0.387	34.85	0.392
9	5	3	34.39	0.384	34.46	0.388
10	3.5	4	19.84	0.083	19.88	0.087
11	3.5	3	20.28	0.139	20.07	0.148
12	3.5	3	21.02	0.131	21.01	0.137
13	2.43934	3.70711	21.32	0.237	20.93	0.244

**Table 2:** Characterization of optimized OST-ME.

DS (nm)	PDI	pH	Osmolarity (mOsm/kg)	EE (%)	DL (%)	ZP (mv)
16.18 ± 0.02	0.09 ± 0.00	6.61 ± 0.99	298.89 ± 1.54	99.15 ± 0.66	3.70 ± 0.53	−1.18 ± 0.97

Note: DS, droplet size; PDI, polydispersity index; EE, entrapment efficiency; DL, drug loading; ZP, zeta potential.

**Table 3:** The different mathematical models’ fitting for OST-ME and OST-Susp.

Mathematical Models									
Formulation	Zero-order		First-order		Higuchi		Korsmeyer–Peppas		
	K(h)	*R* ^2^	K(h)	*R* ^2^	K(h^1/2^)	*R* ^2^	K(h)	n	*R* ^2^
OST-ME	0.77	0.92	0.25	0.86	5.42	0.99	7.62	0.42	0.98
OST-Susp	0.09	0.13	0.51	0.94	1.23	0.38	10.14	0.16	0.14

**Table 4:** The results of pharmacokinetic parameters.

Tissue	Pharmacokinetic Parameters	Unit	OST-ME	OST-Susp
Cornea	C_max_	μg/g	1.56 ± 0.55	0.18 ± 0.21
	T_max_	min	5	5
	T_1/2_	min	28	/
	AUC_0-t_	μg/g·min	19.74	0.7
Conjunctiva	C_max_	μg/g	2.44 ± 0.14	0.15 ± 0.11
	T_max_	min	10	5
	T_1/2_	min	25	/
	AUC_0-t_	μg/g·min	63.96	0.625

Note: AUC, area under curve.

**Table 5:** The OST-ME was optimized by CCD-RSM.

	Levels				
Factors (independent variables)	−1.414	−1	0	1	1.414
X_1_ (oil concentration, %)	2	2.44	3.5	4.56	5
X_2_ (Km range)	2	2.29	3	3.71	4
Responses (dependent variables)	Desirability constraints				
Y_1_: DS (nm)	Minimize				
Y_2_: PDI	Minimize				

Note: DS, droplet size; PDI, polydispersity index.

## Data Availability

Data is contained within the article.
